# Will Guiteau Receive the Benefit of a Doubt?

**Published:** 1881-12

**Authors:** 


### Will Guiteau Receive the Benefit of a Doubt?

If the President's assassin had been a man of some social standing, or had been wealthy, it is probable there would have been no lack of so-called physicians, whose desire for justice would have urged them to testify in the strongest terms that he was insane.

The singular mixture of some viciousness and much foolishness which characterized the former career of the fellow, his fanatical notions concerning himself and his own divine mission, and the statement that there is a crazy streak somewhere in the family, would, in many cases, be amply sufficient reason for sustaining such a plea by abundant medical testimony. The definitions of insanity are often so elastic as to bring within their scope those who have proved themselves vastly more rational than this notorious "crank." It is easy to recall more than one apparently deliberate murder, in which the plea of temporary insanity cheated the gallows of a victim; and it is also worthy of note that if such evil-doers were deemed proper inmates for a madhouse, they usually managed to recover from their malady with a rapidity which was quite astonishing. If Guiteau's cause were not one so universally unpopular, it is probable that this might also be urged with greater effect than it is, in his defense. It was with difficulty, however, that he obtained any counsel and it would, probably, be next to impossible to find men to interest themselves for him. It is generally conceded that the world is better off without such vermin upon it. This is the popular verdict.

But the voice of the people is not always the voice of justice; and if the law was a fair one, by which other men have escaped the death penalty, why should not this one avail himself of it?

It is quite another question as to whether the assassin of our chief magistrate should be allowed under any circumstances to go unpunished. That does not concern us at present. We only contend that it will be especially difficult for this man to secure strict legal justice, and in future years, when resentment will have cooled, and when the account of this trial will be written, the historian may say that the plea of insanity was not urged more strongly partly because frank and fearless doctors were too few.

